# Processing of Mutant β-Amyloid Precursor Protein and the Clinicopathological Features of Familial Alzheimer’s Disease

**DOI:** 10.14336/AD.2018.0425

**Published:** 2019-04-01

**Authors:** Christopher Bi, Stephanie Bi, Bin Li

**Affiliations:** ^1^Washington Institute for Health Sciences, Arlington, VA 22203, USA; ^2^Department of Biochemistry and Molecular & Cellular Biology, Georgetown University Medical Center, Washington DC 20057, USA

**Keywords:** Alzheimer’s disease, APP mutation, APP processing, clinico-pathological features, cellular models, animal models

## Abstract

Alzheimer’s disease (AD) is a complex, multifactorial disease involving many pathological mechanisms. Nonetheless, single pathogenic mutations in amyloid precursor protein (APP) or presenilin 1 or 2 can cause AD with almost all of the clinical and neuropathological features, and therefore, we believe an important mechanism of pathogenesis in AD could be revealed from examining pathogenic APP missense mutations. A comprehensive review of the literature, including clinical, neuropathological, cellular and animal model data, was conducted through PubMed and the databases of Alzforum mutations, HGMD, UniProt, and AD&FTDMDB. Pearson correlation analysis combining the clinical and neuropathological data and aspects of mutant APP processing in cellular models was performed. We find that an increase in Aβ42 has a significant positive correlation with the appearance of neurofibrillary tangles (NFTs) and tends to cause an earlier age of AD onset, while an increase in Aβ40 significantly increases the age at death. The increase in the α-carboxyl terminal fragment (CTF) has a significantly negative correlation with the age of AD onset, and β-CTF has a similar effect without statistical significance. Animal models show that intracellular Aβ is critical for memory defects. Based on these results and the fact that amyloid plaque burden correlates much less well with cognitive impairment than do NFT counts, we propose a “snowball hypothesis”: the accumulation of intraneuronal NFTs caused by extracellular Aβ42 and the increase in intraneuronal APP proteolytic products (CTFs and Aβs) could cause cellular organelle stress that leads to neurodegeneration in AD, which then resembles the formation of abnormal protein “snowballs” both inside and outside of neurons.

With an estimated 5.5 million cases to date, Alzheimer’s disease (AD) is one of the most prevalent neurological dementia disorders in the United States, and the number of cases is projected to rise to over 13 million by 2050 [1]. According to the World Alzheimer Report 2016, there were 46.8 million people living with dementia in 2015, and the number of cases worldwide is projected to reach 131.5 million by 2050 [2]. AD is characterized by the gradual loss of cognitive and behavioral functions, which accounts for 50-70% of dementia cases. Post-mortem examinations of AD patients reveal amyloid plaques composed of β-amyloid (Aβ) peptides and neurofibrillary tangles (NFTs) composed of hyperphosphorylated tau protein, which are thought to be linked to the death of neurons [3]. Approximately 75% of cases are termed sporadic AD with no apparent connection to family history, while the other 25% are related to family history. Among all AD cases, approximately 95% are late onset (age >60 or 65 years), and 5% are early onset (age <65 years). Fewer than 2% of AD cases belong to early-onset familial AD (EOFAD). 10% to 15% of EOFAD are caused by amyloid precursor protein (APP) mutation, and 20% to 70% of EOFAD are caused by presenilin 1 (PS1) mutation; in rare cases, EOFAD can be caused by presenilin 2 (PS2) mutations [4]. Currently, there is no cure for AD — only a few drugs, such as acetylcholinesterase inhibitors and memantine that can temporarily alleviate cognitive and behavioral symptoms [5]. To find an effective treatment strategy, many hypotheses have been proposed to explain the pathogenesis of AD, including the amyloid cascade hypothesis [6], the presenilin hypothesis [7], the APP matrix hypothesis [8], hyperphosphorylation of tau protein [9], neuroinflammation [10], oxidative stress [11], mitochondrial dysfunction [12], endoplasmic reticulum (ER) stress [13], dysregulation of glucose [14], cholesterol and metal metabolism [15], and aberrant cell cycle reentry [16]. As a complex, multifactorial disease, it is reasonable to believe that AD involves a variety of pathological mechanisms. However, familial AD and sporadic AD are largely indistinguishable with respect to symptoms — namely, single pathogenic missense mutations in APP, PS1 or PS2 can cause AD with almost all of the clinical and neuropathological features. Familial AD that is caused by missense mutations of APP can provide a disease model to reveal an important mechanism of pathogenesis in AD.

In this paper, we first briefly describe the function and processing of normal APP as well as the neuropathological features of AD. We then review the clinical and neuropathological characteristics of AD caused by APP missense mutations, mutant APP processing in cellular models, and the histological and behavioral manifestations of transgenic (Tg) animal models. A total of 27 APP missense mutations are included in this review. Next, we analyzed the correlation between clinical and neuropathological data as well as the features of mutant APP processing in cellular models. Finally, based on the analysis results, we propose the “Snowball Hypothesis”: accumulation of extracellular Aβ42 does not directly cause neuronal death; instead, their gradual accumulation causes NFTs formation and organelle stress within neurons. Intracellular APP proteolytic products also aggravate organelle stress. This abnormal intracellular protein accumulation can cause neurons to slowly lose function and finally die.

## The function and processing of normal APP

The APP gene, which is located on chromosome 21 in humans, encodes a type I transmembrane protein that exists in 8 different isoforms, with the three main isoforms being APP695, APP751, and APP770, containing 695, 751, and 770 amino acids, respectively [17]. APP751 and APP770 both contain a 56-amino acid-long Kunitz Protease Inhibitor (KPI) region and are primarily expressed by microglia and meninges cells in the brain, while APP695, the dominant isoform, is primarily expressed by neurons. In fact, it is estimated that KPI-containing isoforms of APP account for less than 14% of the APP produced in the brain, but interestingly, the mRNAs of these isoforms have been observed at elevated levels in AD patients and are thought to be related to Aβ deposition [18].

APP belongs to a group of proteins that includes APP-like protein 1 (APLP1) and APP-like protein 2 (APLP2). They all are type I transmembrane proteins and are processed in a similar fashion, but the Aβ domain is unique to the APP gene. Studies suggest that there are certain redundancies between these proteins. For example, studies demonstrate that mice lacking a single APP family member are viable and fertile, showing certain abnormal physiological changes such as decreased brain size and weight. However, APP/APLP2 and APLP1/APLP2 double-null mice and APP/APLP1/APLP2 triple-null mice show mortality in early development, while APP/APLP1 double-null mice are viable, suggesting that APLP2 is the most important [19, 20].

The APP family is thought to be involved in intercellular adhesion based on the study of certain conserved regions. For example, the extracellular portion of APP has been found to associate with heparin, collagen type I, and laminin. In addition, the E2 domain was found to form antiparallel dimers, and transcellular APP/APP interactions induce presynaptic specializations in co-cultured cells, signifying APP proteins as a class of synaptic adhesion molecules. Through its adhesion properties, APP is also known to possess neural and synaptotropic functions, stimulating short-term neuronal adhesion and long-term neurite growth [21]. The notion that soluble APPsα, a derivative of APP, is essential to neuronal protective functions is supported by its correlation to higher synaptic density and its role in spatial memory [22]. Other functions of APP and its fragments, such as axon pruning and degeneration [23], intracellular signaling, and apoptosis, are still ambiguous [21]

In non-polarized cells, APP is first synthesized in the ER and is then transported to the Golgi apparatus and trans-Golgi network (TGN), in which it is found in the highest steady-state concentration. A portion of the APP (estimated to be approximately ~10%) is destined for the plasma membrane, and it is posttranslationally modified by N-linked and O-linked glycosylation, palmitoylation, and tyrosine sulfation, among others. APP that reaches the plasma membrane without cleavage within minutes is rapidly internalized in endosomes. A fraction of these endosomes is recycling endosomes that release it back to the plasma membrane, while others undergo lysosomal degradation. α-Secretases such as ADAM9, ADAM10, and ADAM17, belonging to the “disintegrin and metalloprotease” family, are thought to reside primarily at the cell membrane, cleaving APP to form the APPsα derivative and a carboxyl terminal fragment (α-CTF or C83) in the non-amyloidogenic pathway. α-CTFs are further cleaved by γ-secretase to form a truncated Aβ peptide called p3 and the APP intracellular domain (AICD). β-Secretases, one of which is known as β-secretase 1 (BACE1), are thought to be localized in the acidic environments of the TGN, Golgi, and endosomes and cut APP at the β sites in the amyloidogenic pathway, producing the ectodomain APPsβ. The carboxyl terminal fragment of APP that are leftover, known as β-CTF (C99), remains plasma membrane-bound and is further cleaved by γ-secretase to form Aβ and AICD. APP processing in neurons is thought to be similar in non-polarized cells, but one difference is that after leaving the neuronal TGN, APP is transported to axons and dendrites [19, 24].

### Neuropathological features of Alzheimer’s disease

AD is typically diagnosed in a post-mortem examination of the brain. Macroscopic features of an AD brain include cortical atrophy that mainly affects the medial temporal lobes. Due to cortical atrophy, certain phenotypes, such as lateral ventricular and temporal horn dilatation, can manifest themselves. Microscopic features include neurofibrillary tangles (NFTs), amyloid plaques, cerebral amyloid angiopathy (CAA), glial responses, neuron and synapse loss, granuovacuolar degeneration, Hirano bodies and Lewy bodies [25].

NFTs are primarily composed of paired helical filaments, which are constructed from aberrant hyperphosphorylated tau protein [26]. NFTs are classified into three main categories: 1) pre-NFTs appearing in the cytoplasm of otherwise-normal neurons; 2) mature intraneuronal NFTs consisting of cytoplasmic filaments that displace the nucleus towards the periphery of the soma in neurons; and 3) “ghost” NFTs, which are characterized by neuronal death and cytoplasmic staining of tau. NFT stage advancement parallels neuron and synapse loss. The correlation between the progression of NFTs and the development of dementia symptoms has been firmly established [27].

Amyloid plaques are mainly composed of the extracellular accumulation of Aβ40 and Aβ42 derived from APP after cleavage by β- and γ-secretases. Aβ42 is considered to be more insoluble and has a higher chance of polymerization and therefore is typically more abundant in plaques, while Aβ40 is investigated as the primary constituent in CAA. Amyloid plaques can be categorized into diffuse and dense-core plaques. A drastic difference between amyloid plaques and NFTs is that amyloid plaques occur mainly in the isocortex. Braak and Braak distinguished between three stages of amyloid plaques: Stage 1, occurring mainly in the isocortex; Stage 2, in the limbic region; and Stage 3, in the subcortical layer [28]. Despite this, clinicopathological studies showed that the amyloid burden does not correlate with the severity or the duration of dementia [27].

Although Alzheimer’s disease is characterized by NFTs and amyloid plaques, some 38.2% of non-demented people may qualify in the “intermediate” or “high likelihood of AD” categories of the National Institute on Aging (NIA) - Reagan criteria indicated by the presence of moderate neuritic plaques and NFTs in limbic regions [29]. In fact, certain asymptomatic AD individuals seem resistant to NFTs and amyloid burden, perhaps contradicting the correlation between NFTs/amyloid and neuronal death. Bussièreet *et al.* found that a considerable number of neurons containing mature intraneuronal NFTs can survive for almost two decades [30]. Other evidence also suggests that the presence of NFTs does not directly cause neuronal dysfunction [31]. Therefore, we might not say that the accumulation of Aβ and NFTs are “toxic” — rather, they are an increasing burden to neurons.

CAA, identified by deposits of Aβ in leptomeningeal arteries, small and medium-sized arterioles, and cortical capillaries of the central nervous system, is found in ~80% of AD patients. Leptomeningeal arteries are typically affected more than cortical arteries, and CAA can lead to complications such as lobar hemorrhages [25].

Activated microglia and astrocytes are commonly correlated negatively with dense-core plaques, but Serrano-Pozo *et al.* found that glial responses also correlated positively with NFT burden. This finding argued against the stereotypical relationship between glial cells and amyloid plaques and suggested that reactive glia might contribute to the ongoing neurodegeneration [32].

Cortical atrophy is mainly caused by neuronal loss. NFTs levels parallel neuronal loss in regional and laminar patterns, but in certain regions, neuronal loss has been found to be greater, suggesting neuronal loss as a better measure of cognitive damage [33]. Synaptic loss has also matched neuronal loss in the same spatiotemporal and laminar patterns, but synaptic loss can exceed the existing neuronal loss, which suggests that loss of neuronal function precedes neuronal death [34].

Hirano bodies and vascular degeneration may also appear as lesions in the hippocampal formation. Studies have suggested that these bodies are associated with ER stress [35, 36]. Lewy bodies are abnormal aggregates of α-synuclein that develop inside neurons in Parkinson's disease (PD), commonly associated with Lewy body dementia. Approximately 70% of patients with sporadic AD and some familial AD display Lewy body-like pathology in the amygdala and limbic structures [37, 38].

### Familial AD caused by missense mutations in APP

A comprehensive literature search of the PubMed, Alzforum, HGMD, UniProt, and AD&FTDMDB databases was conducted; 69 missense mutations were reported in the above databases. Among them, 36 mutations (A201V, A235V, D243N, E246K, T276S, V287G, E296K, P299L, R328W, V340M, R468H, A479S, K496Q, A500T, K510N, Y538H, V562I, E599K, T600M, S614G, P620A, P620L, N660Y, T663M, E665D, H677R, G708G, G709S, V710G, A713V, I718L, T719N, L720S, M722K, H733P, and A741S) are not pathogenic or are unclear; 7 mutations (E693N, L705V, A713T, T714A, I716M, T719P, and L723R) are pathogenic, but their effects on APP processing have not been studied in cellular models; 25 APP pathogenic missense mutations (KM670/671NL, A673V, D678H, D678N, E682K, K687N, A692G, E693del, E693G, E693K, E693Q, D694N, T714I, V715A, V715M, I716F, I716T, I716T, V717F, V717G, V717I, V717L, L723P, K724N, K724M) and 1 protective mutation (A673T) are included in this review. All of these mutations are clustered around α-, β- and particular γ-secretase cleavage sites that influence APP proteolysis. APP pathogenic missense mutations cause familial AD with dominant inheritance, except for the A673V mutation, which shows a recessive Mendelian pattern of inheritance [39]. The mean ages of onset and death were calculated based on available references, and the pathological findings from different references are summarized. We give priority to neuronal cell models when making a judgment on the effects of APP mutations in different cell lines. Tg animal model studies with a single pathogenic APP mutation are also discussed in this review.

#### KM670/671NL (Swedish) mutation

The mean age of onset was 53.8 years (n=70), and the mean age of death was 62.3 years (n=42). Brain imaging examinations include computed tomography (CT), magnetic resonance imaging (MRI) and single photon emission computed tomography (SPECT), showing cortical atrophy and cerebral blood flow reduction. The reported neuropathological characteristics included NFTs, amyloid plaques, CAA, and Lewy bodies [40 - 47]. Studies of APP processing in cellular models showed that intracellular Aβ increased compared to the WT control; α-CTF levels remained constant; β-CTF increased; total secreted Aβ, Aβ40 and 42 increased; and the ratio of Aβ42/Aβ40 did not change [48-53]. The APP23 mouse model carrying the M670/671NL mutation (APP 751) driven by the murine Thy1 promoter had 7-fold higher expression of mutant human APP compared to endogenous mouse APP. Histological studies showed no NFTs, while amyloid plaques appeared in the cortex and hippocampus at 6 months, and CAA appeared at 19 months. Activation of microglia appeared at 14 months, and neuronal loss (14-28%) was found in the CA1 region of the hippocampus in 14- to 18-month-old mice. Memory defects were found at 3 months [54-60]. The TAS10 (thy1-APPswe) mouse model carries the KM670/671NL mutation (APP695) driven by the murine Thy1 promoter. Histological studies showed no NFTs, but phosphorylated tau appeared in the hippocampus and cortex at 24 months, and amyloid plaques appeared in the cortex and hippocampus at 12 months. Furthermore, activation of microglia and astrocytes appeared at 6 months. While no neuronal loss was found, synaptic loss appeared at 24 months, and the number of lysosomes increased in neurons of the dentate gyrus at 6 months. Memory defects were found at 6 months [61]. The Tg-Swe mouse model carries the KM670/671NL mutation (APP695) driven by the murine Thy1 promoter. Histological studies showed no NFTs, amyloid plaques appeared in the cortex and hippocampus at 12 months, and CAA appeared at 12 months. Intraneuronal Aβ accumulation occurred at 6 months, activation of microglia and astrocytes appeared at 12 months. No neuronal loss was found, and memory defects are not known [62-64]. The APPSwe (line C3-3) mouse model carries a chimeric mouse/human APP695 containing a "humanized" Aβ domain with the KM670/671NL mutation driven by the mouse prion protein promoter. These mice showed 3-fold higher expression of mutant human APP compared to endogenous mouse APP. Histological studies showed that amyloid plaques appeared in the cortex and hippocampus at 18 months, but memory defects were not found [65, 66]. The APPSwe-NSE mouse model carries human APP695 containing the KM670/671NL mutation driven by the neuron-specific enolase (NSE) promoter. Histological studies showed widespread, intensive staining of Aβ42 in the neurons of the cortex and hippocampus at 12 months but no amyloid plaques. The number of TUNEL-stained nuclei increased. Biochemical studies showed that Aβ42 and tau phosphorylation increased at 12 months. Memory defects were found at 12 months [67]. The Tg2576 mouse model carries human APP695 containing the KM670/671NL mutation driven by the hamster prion protein. Histological studies showed that there were no NFTs, while amyloid plaques appeared in the cortex and hippocampus at 11 months. Activation of microglia appeared at 10 months, but no neuronal loss was found. Dendritic spine loss appeared at 4.5 months in the CA1 region of the hippocampus, and memory defects were found at 9 months [68, 69].

#### A673T (Icelandic) mutation

This mutation has been reported to have protective effects. The age of onset for 1 dementia case was 104 years. The reported neuropathological characteristics included NFTs, no amyloid plaques, and mild CAA. Studies of APP processing in cellular models showed that β-CTF decreased compared with WT control; total secreted Aβ, Aβ40 and Aβ42 all decreased; the ratio of Aβ42/Aβ40 all did not change; Aβ40 and Aβ42 polymerization decreased [70-74]. There is no Tg animal model available for this mutation.

#### A673V mutation

Only one case was reported, the age of onset was 36.0 years, and the age of death was 46 years. Brain imaging examination (MRI) showed cortical atrophy. The reported neuropathological characteristics included NFTs, amyloid plaques, CAA, activation of microglia and astrocytes, and neuronal loss [39, 75]. Studies of APP processing in cellular models showed that β-CTF increased compared to WT control; total secreted Aβ, Aβ38, Aβ40 and Aβ42 all increased; the ratio of Aβ42/Aβ40 showed no change; and Aβ40 polymerization increased [39, 72, 73]. There is no Tg animal model available for this mutation.

#### D678H (Taiwanese) mutation

Only one case was reported, and the age of onset was 51.0 years. Brain imaging examinations (CT and SPECT) showed cortical atrophy and reduced cerebral blood flow. Neuropathological reports are not available. Studies of APP processing in cellular models showed that intracellular Aβ did not change compared with WT control, whereas β-CTF, total secreted Aβ, Aβ40 and Aβ42, and the ratio of Aβ42/Aβ40 all increased. Aβ40 polymerization increased, but Aβ42 aggregation decreased, while internalization of APP increased and degradation of APP in lysosomes increased [76, 77]. There is no animal model available for this mutation.

#### D678N (Tottori) mutation

The mean age of onset was 58.8 years (n=3), and the mean age of death was 70.3 years (n=3). Brain imaging examinations (MRI and SPECT) showed cortical atrophy and cerebral blood flow reduction. Neuropathological reports are not available. Studies of APP processing in cellular models reported that intracellular Aβ showed no change compared with WT control; β-CTF showed no change; and the total secreted Aβ, Aβ40, Aβ42, and the ratio of Aβ42/Aβ40 all showed no change. Aβ42 polymerization increased [78-80]. There is no animal model available for this mutation.

#### E682K (Leuven) mutation

The mean age of onset was 61.0 years (n=2), and the age of death was 83 years (n=1). Brain imaging examination (MRI) showed hippocampal atrophy. Neuropathological reports are not available. Studies of APP processing in cellular models showed that α-CTF did not change compared with WT control; β-CTF, total secreted Aβ, Aβ40 and 42, and the ratio of Aβ42/Aβ40 all increased [81]. There is no animal model available for this mutation.

#### K687N mutation

The mean age of onset was 56.3 years (n=3), and the mean age of death was 68.3 years (n=3). Brain imaging examination (MRI) showed cortical atrophy. Neuropathological reports are not available. Studies of APP processing in cellular models showed that membrane-associated APP increased compared to WT control; α- and β-CTF decreased; total secreted Aβ, Aβ40, Aβ42, and the ratio of Aβ42/Aβ40 increased; and resistance to proteolytic degradation with neprilysin increased [82]. There is no animal model available for this mutation.

#### A692G (Flemish) mutation

The mean age of onset was 46.0 years (n=15), and the mean age of death was 54.6 years (n=15). The reported neuropathological characteristics included cortical atrophy, hemorrhagic infarction, NFTs, amyloid plaques, CAA, activation of microglia and astrocytes, and neuronal loss [46, 83-85]. Studies of APP processing in cellular models showed that α-CTF did not change compared to WT control; β-CTF increased; total secreted Aβ, Aβ40, 42 and the ratio of Aβ42/Aβ40 all increased; Aβ40 and 42 polymerizations decreased; and the resistance to proteolytic degradation with neprilysin and insulin-degrading enzyme increased [81, 86-92]. There is no animal model available for this mutation.

#### E693del (Osaka) mutation

The mean age of onset was 49.7 years (n=7), and the mean age of death was 59.5 years (n=4). Brain imaging examinations (MRI and SPECT) showed cortical atrophy and cerebral blood flow reduction. [18F]-Fluorodeoxyglucose positron emission tomography (FDG-PET) showed reduced glucose metabolism (hypometabolism) in the cerebral cortex. PET amyloid imaging showed less Aβ deposition than in typical idiopathic AD brains. Neuropathological reports are not available. Studies of APP processing in cellular models showed that intracellular Aβ increased compared to WT control; total secreted Aβ, Aβ40 and Aβ42 decreased; the ratio of Aβ42/Aβ40 did not change; Aβ40 and 42 polymerizations increased; and the resistance to proteolytic degradation with neprilysin and insulin-degrading enzyme increased [93-95]. The APP E693Δ-Tg (Osaka) mouse model carrying the Osaka mutation (APP695) driven by the mouse prion promoter expressed similar amounts of mutant human APP and endogenous mouse APP. Histological studies showed no NFTs, but abnormal tau phosphorylation was found at 8 months. There were no amyloid plaques, while intraneuronal Aβ accumulation in the hippocampus and cerebral cortex occurred at 8 months. Activation of microglia appeared at 12 months; activation of astrocytes appeared at 18 months; neuronal loss in the CA3 region of the hippocampus was found at 18 months; synaptic loss in the CA3 region appeared at 8 months. Memory defects were found at 8 months [96, 97]. The OSK-KI mouse model carrying the Osaka mutation was generated by knock-in of this mutation into endogenous mouse APP. The levels of APP expression in homozygous and heterozygous mice were similar to those of endogenous APP in non-knock-in mice. Histological studies showed no NFTs, but abnormal tau phosphorylation was found in homozygotes at 8 months. There were no amyloid plaques, while intraneuronal Aβ accumulation in the hippocampus and cerebral cortex occurred in homozygotes at 8 months, and heterozygotes showed only slight Aβ accumulation at 24 months. Activation of microglia and astrocytes appeared at 12 months in homozygotes; gliosis was not found in heterozygotes; neuronal loss in the hippocampus and entorhinal cortex was found at 24 months; synaptic loss appeared at 8 months in homozygotes and at 24 months in heterozygotes. Memory defects in homozygotes were found at 4 months [98].

#### E693G (Arctic) mutation

The mean age of onset was 57.0 years (n=43), and the mean age of death was 65.5 years (n=15). Brain imaging examinations (MRI and SPECT) showed cortical atrophy and cerebral blood flow reduction. The reported neuropathological characteristics included NFTs, amyloid plaques, intracellular Aβ immunoreactivity, CAA, activation of astrocytes, and neuronal loss [44, 99-103]. Studies of APP processing in cellular models showed that total secreted Aβ38 increased compared to the WT control; Aβ40 did not change; Aβ42 and the ratio of Aβ42/Aβ40 decreased; Aβ40 and 42 polymerizations increased; and the resistance to proteolytic degradation with neprilysin increased [86, 91, 103, 104]. The TgAPParc mouse model carrying the E693G mutation (APP695) driven by the murine Thy1.2 promoter had 3- to 7-fold higher expression of mutant human APP compared to endogenous mouse APP. Histological studies showed no NFTs, but strong intracellular Aβ immunoreactivity in the hippocampus and cortex appeared at 3 months. A diffuse extracellular immunoreactivity appeared in some brain areas at 4 months; plaque-like structures in the subiculum appeared at 6 months; dense Aβ plaques with Congo red birefringence appeared in the subiculum at 9 months. Biochemical studies showed that Aβ40 and Aβ42 increased at 12 months. Memory defects were found at 15 months [105, 106].

#### E693K (Italian) mutation

The age of dementia onset is not available, the mean age of first brain hemorrhage was 49.9 years (n=7), and the mean age of death was 60.0 years (n=7). Brain imaging examinations (CT and MRI) showed cerebral hemorrhages, multi-infarct encephalopathy, and leukoaraiosis. The reported neuropathological characteristics included hemorrhages, no NFTs, amyloid plaques, and CAA [102, 107]. Studies of APP processing in cellular models showed that the secreted Aβ38 increased compared to the WT control; secreted Aβ40 did not change; Aβ42 and the ratio of Aβ42/Aβ40 decreased; Aβ42 polymerization increased; and the resistance to proteolytic degradation with neprilysin increased [86, 91, 104]. There is no animal model available for this mutation.

#### E693Q (Dutch) mutation

The age of dementia onset is not available, the mean age of first brain hemorrhage was 53.7 years (n=6), and the mean age of death was 57.9 years (n=11). Brain imaging examinations (CT and MRI) showed cerebral hemorrhages. The reported neuropathological characteristics included hemorrhages, no NFTs, amyloid plaques, and CAA [102, 108-114]. Studies of APP processing in cellular models showed that the total secreted Aβ and Aβ38 did not change compared to the WT control; secreted Aβ40 and Aβ42 decreased; the ratio of Aβ42/Aβ40 decreased; Aβ40 polymerization increased; and the resistance to proteolytic degradation with neprilysin increased [86, 91, 104]. The APP Dutch mouse model carries the E693Q mutation (APP 751) driven by the murine Thy1 promoter. Histological studies showed no NFTs and no amyloid plaques. CAA appeared at 22 months; activation of microglia and astrocytes appeared at 29 months; hemorrhage occurred at 29 months [115]. There is another transgenic mouse model carrying the E693Q mutation (APP751) driven by the murine Thy1 promoter. Histological studies showed no NFTs and no amyloid plaques. Intraneuronal Aβ was detected at 2 months; intraneuronal lysosomal accumulation of CTFs and lysosomal abnormality appeared at 12 months; CAA appeared at 12 months; loss of cholinergic neurons and GABAergic interneurons were found at 12 months; activation of microglia and astrocytes appeared at 12 months [116, 117].

#### D694N (Iowa) mutation

The mean age of dementia onset was 58.3 years (n=7), the mean age of first brain hemorrhage was 43.8 years (n=9), and the mean age of death was 65.3 years (n=11). Brain imaging examinations (CT and MRI) showed cerebral hemorrhages. The reported neuropathological characteristics included NFTs, amyloid plaques, CAA, and astrocyte activation [118-122]. Studies of APP processing in cellular models showed that α-CTF, total secreted Aβ, Aβ38, Aβ40, Aβ42, and the ratio of Aβ42/Aβ40 all unchanged compared to the WT control; Aβ40 and Aβ42 polymerization increased [86, 123]. There is no animal model available for this mutation.

#### T714I (Austrian) mutation

The mean age of onset was 34.0 years (n=3), and the mean age of death was 46.3 years (n=3). The reported neuropathological characteristics included NFTs, amyloid plaques, CAA, activation of microglia and astrocytes, and neuronal loss [124-127]. Studies of APP processing in cellular models showed that the intracellular Aβ, α- and β-CTF levels increased compared to the WT control; secreted Aβ40 decreased; Aβ42 and the ratio of Aβ42/Aβ40 increased [125-127]. The heterozygous APP-Au5 mouse model carrying the T714I mutation (APP695) driven by the PDGF promoter showed expression levels of one-tenth of endogenous mouse APP levels. Brain volumes of APP-Au5 mice were significantly reduced on volumetric MRI at 12 months. Histological studies showed no NFTs and no amyloid plaques, but intraneuronal Aβ was observed in the perikaryon of the pyramidal neurons of the subiculum and of the CA1 and CA2 hippocampal regions at 6 months. Neuronal loss was not found. Memory defects were not found [128].

#### V715A (German) mutation

The mean age of onset was 50 years (n=5), and the mean age of death was not available. Brain imaging examination (PET) showed parieto-occipital hypometabolism. Neuropathological reports are not available [129, 130]. Studies of APP processing in cellular models showed that α- and β-CTF levels were comparable to those in WT controls; secreted Aβ40 decreased; secreted Aβ42 and the ratio of Aβ42/Aβ40 increased [127, 129, 131]. There is no animal model available for this mutation.

#### V715M (French) mutation

The mean age of onset was 47.8 years (n=5), and the mean age of death was 55.8 years (n=5). Brain imaging examinations (MRI and PET) showed cortical atrophy and hypometabolism. Neuropathological reports are not available [53, 132]. Studies of APP processing in cellular models showed that intracellular Aβ decreased compared to WT control; α-CTF level increased; β-CTF levels remained constant; total secreted Aβ and Aβ40 decreased; secreted Aβ42 and the ratio of Aβ42/Aβ40 increased [53, 127, 131]. There is no animal model available for this mutation.

#### I716F (Iberian) mutation

The mean age of onset was 36.0 years (n=4), and the mean age of death was 36.7 years (n=3). Brain imaging examinations (MRI and SPECT) showed cortical atrophy and cerebral blood flow reduction. The reported neuropathological characteristics included NFTs, amyloid plaques, CAA, activation of microglia and astrocytes, neuronal loss, and Lewy bodies [133-135]. Studies of APP processing in cellular models showed that the membrane-associated APP increased compared to the WT control; β-CTF increased; total secreted Aβ decreased; Aβ38 did not change; Aβ40 decreased; Aβ42 and the ratio of Aβ42/Aβ40 increased [126, 134, 136]. There is no animal model available for this mutation.

#### I716T mutation

Only one case was reported, the age of onset was 36.0 years, and the age of death was 43 years. Neuropathological reports are not available [137]. Studies of APP processing in cellular models showed that the levels of membrane-associated APP and β-CTF did not change compared to the WT control; total secreted Aβ did not change; Aβ38 increased; Aβ40 decreased; Aβ42 and the ratio of Aβ42/Aβ40 increased [136]. There is no animal model available for this mutation.

#### I716V (Florida) mutation

The mean age of onset was 52.7 years (n=3), and the mean age of death was 60.0 (n=1). Brain imaging examination (MRI) showed cortical atrophy. Neuropathological reports are not available [138]. Studies of APP processing in cellular models showed that the levels of membrane-associated APP did not change compared to the WT control; α-CTF levels decreased; β-CTF levels remained constant; total secreted Aβ did not change; Aβ38 increased; Aβ40 did not change; Aβ42 and the ratio of Aβ42/Aβ40 increased [127, 136, 138]. There is no animal model for this mutation.

#### V717F (Indiana) mutation

The mean age of onset was 42.1 years (n=14), and the mean age of death was 47.9 years (n=14). The reported neuropathological characteristics included NFTs, amyloid plaques, CAA [46, 139-142]. Studies of APP processing in cellular models showed that the α-CTF level increased compared to the WT control; Aβ40 decreased; Aβ42 and the ratio of Aβ42/Aβ40 increased [104, 126, 143-146]. The PDAPP (line 109) mouse model carrying the V717F (containing APP introns 6-8, allowing expression of mRNAs for human APP695, 751, 770) mutation driven by the platelet-derived growth factor (PDGF)-β promoter had 10-fold expression of mutant human APP compared to endogenous mouse APP. Histological studies showed no NFTs, but phosphorylated tau was found in dystrophic neurites at 14 months. Amyloid plaques appeared in the cortex and hippocampus at 6 months; activation of microglia and astrocytes appeared at 13 months; synaptic loss was found in the dentate gyrus at 13 months. Biochemical studies showed that the total Aβ (mainly Aβ42) concentration in the hippocampus was 500-fold greater at 18 months than at 4 months. Memory defects were found at 3 months [147-149].

#### V717G mutation

The mean age of onset was 56.0 years (n=15), and the mean age of death was 60.7 years (n=5). Brain imaging examination (MRI) showed cortical atrophy. The reported neuropathological characteristics included NFTs, amyloid plaques, and CAA [150-154]. Studies of APP processing in cellular models showed that the level of α-CTF decreased compared to the WT control; Aβ40 decreased; Aβ42 and the ratio of Aβ42/Aβ40 increased [144, 145]. There is no animal model available for this mutation.

#### V717I (London) mutation

The mean age of onset was 50.2 years (n=54), and the mean age of death was 62.1 years (n=53). The reported neuropathological characteristics included NFTs, amyloid plaques, CAA, and Lewy bodies [46, 145, 155-162]. Studies of APP processing in cellular models showed that the intracellular Aβ40 increased compared to the WT control; the levels of membrane associated APP, α-CTF, β-CTF all increased; total secreted Aβ decreased; Aβ38 increased; Aβ40 decreased; Aβ42 and the ratio of Aβ42/Aβ40 increased [48, 89, 126, 127, 134, 138, 144-146, 163, 164]. The APP(V717I) mouse model carrying the V717I mutation (APP695) driven by the murine Thy1 promoter had 5-fold greater expression of mutant human APP compared to endogenous mouse APP. Histological studies showed no NFTs, but dystrophic neurites containing hyperphosphorylated tau appeared at 16 months. Amyloid plaques appeared in the cortex and subiculum at 10 months; CAA appeared at 15 months; activation of microglia appeared at 10 months; neuronal loss was not found. Memory defects were found at 6 months [165, 166]. The APP(V642I)KI mouse model was generated by knocking the V717I mutation into exon 17 of the mouse APP gene using homologous recombination and the Cre-loxP system. The levels of APP expression in heterozygous mutant mice were similar to those of endogenous APP in WT mice. Histological studies showed no NFTs, no amyloid plaques, and no neuronal loss. A biochemical study showed that the ratio of Aβ42/Aβ40 increased at 29 months. Memory defects were found at 27 months [167].

#### V717L (Indiana-2) mutation

The mean age of onset was 44.2 years (n=28), and the mean age of death was 57.3 years (n=17). Brain imaging examinations (MRI and SPECT) showed cortical atrophy and reduced cerebral blood flow. The reported neuropathological characteristics included NFTs and amyloid plaques [140, 168-173]. Studies of APP processing in cellular models showed that the α- and β-CTF levels increased compared to the WT control; Aβ40 decreased; Aβ42 and the ratio of Aβ42/Aβ40 increased [127]. There is no animal model available for this mutation.

#### L723P (Australian) mutation

The mean age of onset was 53.6 years (n=5). One case died at age 53.0. Brain imaging examinations (CT) showed cortical atrophy. Neuropathological reports are not available. Studies of APP processing in cellular models showed that Aβ42 and the ratio of Aβ42/Aβ40 increased compared to the WT control [46, 174]. There is no animal model available for this mutation.

#### K724M mutation

The mean age of onset was 46.6 years (n=5), and the mean age of death was 54.3 years (n=3).

Brain imaging examination (MRI) showed cortical atrophy. Neuropathological reports are not available. Studies of APP processing in cellular models showed that Aβ40 did not change compared to WT control; Aβ42 and the ratio of Aβ42/Aβ40 increased [175]. There is no animal model available for this mutation.

#### K724N (Belgian) mutation

The mean age of onset was 53.5 years (n=2), and the mean age of death was 57.5 years (n=2). One case suffered an aneurysmal subarachnoid hemorrhage at age 50 years. Brain imaging examination (PET) showed cortical hypometabolism and increased Aβ deposition compared to control individuals. Neuropathological reports are not available. Studies of APP processing in cellular models showed that the α- and β-CTF levels did not change compared to the WT control; Aβ38 increased; Aβ40 decreased; Aβ42 and the ratio of Aβ42/Aβ40 increased [163]. There is no animal model available for this mutation.

**Figure 1.**
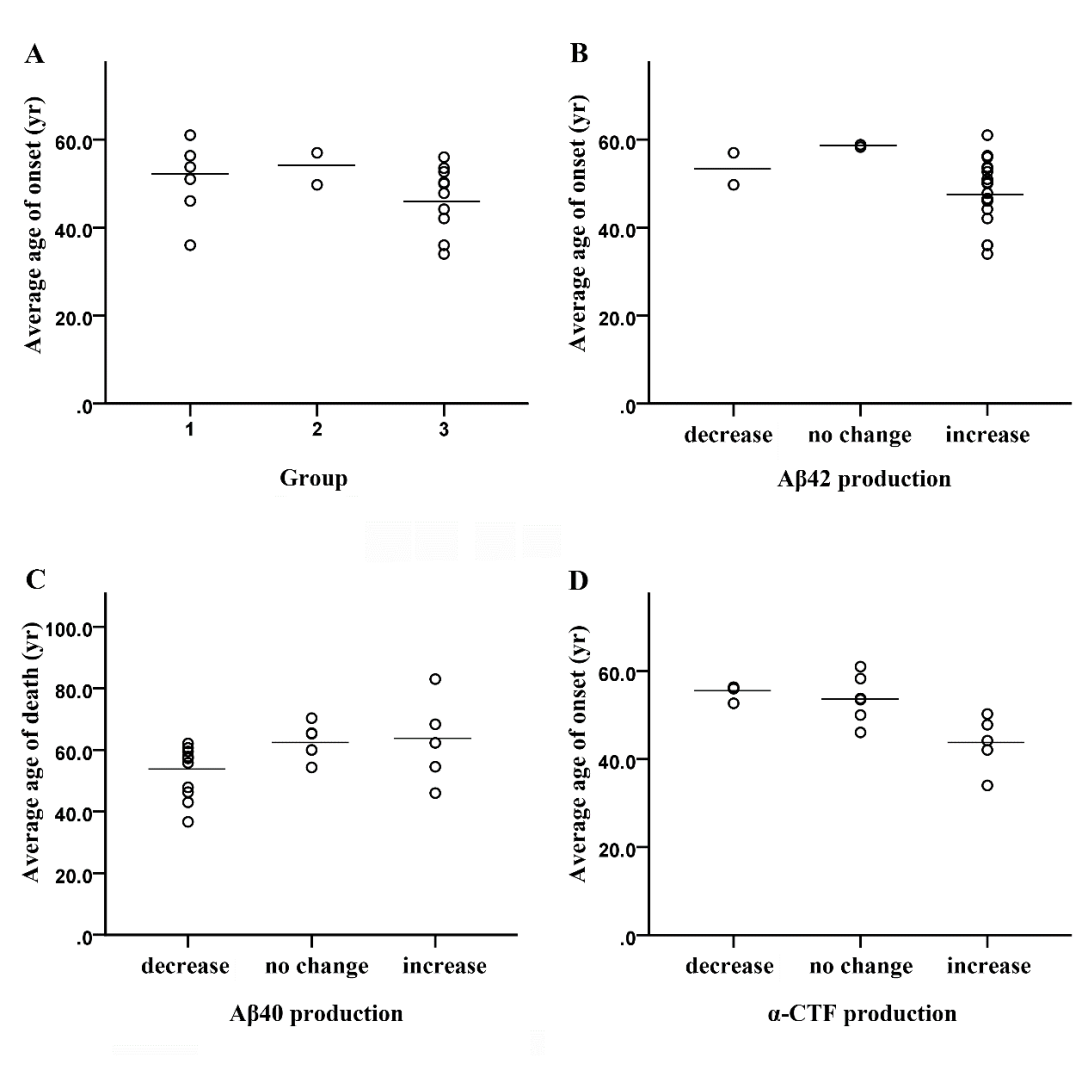
The correlation analysis. (**A**) The average age of AD onset in the three mutation Groups [8]. (**B**) Increased Aβ42 production tends to have an earlier age of AD onset (r = -0.328, p = 0.127). (**C**) Aβ40 production has a significantly positive correlation with age of death (r = 0.440, p = 0.041). (**D**) The intracellular α-CTF production has a significantly negative correlation with age of AD onset (r = -0.661, p = 0.010). Bars represent the mean value of each group.

### Summary and discussion

### The effects of Aβ42 and Aβ40

According to the patterns of total Aβ, Aβ40, Aβ42 secreted from cellular models, Hunter and Brayne divided these mutations into three groups: Group 1 shows increases in total secreted Aβ, Aβ40, Aβ42 and the ratio of Aβ42/Aβ40, and includes KM670/671NL, A673V, D678N, E682K, K687N, and A692G; Group 2 shows reductions in total secreted Aβ, Aβ40, Aβ42 and the ratio of Aβ42/Aβ40, and includes E693del, E693G, E693K, and E693Q; Group 3 shows reductions in total secreted Aβ and Aβ40 combined with increased Aβ42 and the ratio of Aβ42/Aβ40, and include T714I, V715A, V715M, I716F, I716V, V717F, V717G, V717I, V717L, and K724N [8]. The average age of AD onset in Group 1 (6 mutations) is 50.7 ± 8.8 years (mean ± SD), in Group 2 (2 mutations) is 53.4 ± 5.1 years, and in Group 3 (10 mutations) is 46.6 ± 7.4 years (Fig. 1). Although one-way ANOVA does not show significant differences among the three groups, Group 3 (Aβ42 increases only) has a tendency toward an earlier average age of AD onset, and Group 2 (decreases in all Aβs) has a later age of AD onset.

To reveal the correlation between clinical and neuropathological data as well as the features of mutant APP processing in cellular models, Pearson correlation analyses were performed with IBM SPSS software. The appearance of certain pathological features (such as NFTs, amyloid plaques, CAA, etc.) was set as 1, and no appearance was set as 0. An increase in APP proteolytic products compared to the WT control was set as 1, no change as 0, and a decrease as -1. The analysis showed that increased Aβ42 production tends to have an earlier age of AD onset (r = -0.328, p = 0.127). Aβ40 production has a significantly positive correlation with age of death (r = 0.440, p = 0.041) and an insignificantly positive correlation with age of AD onset (r = 0.334, p = 0.128) (Fig. 1). It has been widely accepted that the longer form, Aβ42, is the most amyloidogenic form of the peptide and plays a critical role in AD pathogenesis [176], whereas the shorter Aβ40, which is predominantly associated with CAA, may have a protective effect against Aβ42. Both *in vivo* and *in vitro* studies have confirmed this protective effect and showed short Aβs, such as Aβ38 and Aβ40, could directly interact with Aβ42 to interfere with either the aggregate structure or the kinetics of aggregation to attenuate the deleterious effects of Aβ42 [177, 178].

The correlation analysis also shows that Aβ42 production and the ratio of Aβ42/Aβ40 have significantly positive correlations with the presence of NFTs (r = 0.740, p = 0.004 and r = 0.679, p = 0.011, respectively). The results suggest that the accumulation of extracellular Aβ42 could trigger the formation of intracellular NFTs. As summarized in review papers, clinical studies found that NFTs appeared following amyloid deposition in Down syndrome (carrying an extra copy of APP) and an increase in CSF tau following amyloid deposition in early onset cases with inherited mutations of APP and presenilin. Both cellular and mouse models suggested that the damaging effects of extracellular Aβ on neurons occur through the intracellular tau protein [176, 179-182]. The fact that the progress of dementia symptoms in AD is highly correlated with the development of NFTs rather than amyloid deposition also supports this view [25, 183]. The appearance of NFTs is dependent on the susceptibility of neurons [184-186]. The role of proteasome and autophagy-lysosome systems are to maintain the balance between protein synthesis and protein clearance. Proteasomal stress, which occurs in AD and mild cognitive impairment brains, can alter this balance. Farizatto *et al.* found that extracellular Aß42 could damage the function of proteasome and autophagy-lysosome systems in cultured hippocampal slices, causing an increase in tau phosphorylation [187].

### The effects of intracellular APP proteolytic products

Although the APP mutations in Group 2 can cause decreases in Aβ production in cellular models compared to WT APP, patients with E693G, E693K, and E693Q mutations still showed extracellular amyloid deposition, CAA and typical clinical features of AD. This condition is probably related to the increased Aβ polymerization and resistance to proteolytic degradation for Group 2 mutations. Additionally, cellular model studies indicated that intracellular Aβ increased in Group 2 mutations, especially in cases in which the E693del mutation did not have amyloid deposition but showed progressive cognitive defects [93, 94]. It has been suggested that intracellular Aβ has a critical role in AD [95].

Among the 15 Tg animal models discussed in this review, 11 Tg mouse lines with Aβ deposition completed the cognitive examinations, in which 9 (81.8%) Tg mouse lines (APP23, TSA10, APPSwe-NSE, Tg2576, APP E693Δ-Tg, OSK-KI, PDAPP line 109, APP(V717I), and APP(V642I)KI) showed that memory defects appeared prior to Aβ deposition. Only 2 (18.2%) Tg mouse lines (TgAPParc and APPSwe) showed memory defects after Aβ deposition or no memory defects after Aβ deposition. This fact suggests that amyloid plaque may not be directly responsible for neuronal dysfunction. On the other hand, 5 Tg mouse lines that had completed the cognitive examinations were examined for intracellular Aβ, and 4 (80.0%) of them (TAS10, APPSw-NSE, APP E693Δ-Tg, TgAPParc) showed that intracellular Aβs appeared in advance of or at the same time as memory defects, only 1 (20.0%) Tg mouse line (OSK-KI) showed intracellular Aβs appeared after memory defects. These results may be explained by the accumulation of intracellular Aβ rather than extracellular Aβ deposition causing neuronal dysfunction in advance.

A large number of studies on post-mortem brain samples with AD and Down syndrome have provided evidence for the presence of Aβ within neurons. Studies have shown that the accumulation of intraneuronal Aβ is an early event in the progression of AD, preceding the formation of extracellular Aβ deposits [188]. A neuropathological study on AD cases with the E693G mutation suggested that intracellular Aβ oligomers are more neurotoxic than extracellular Aβ deposition [100]. A transgenic mouse model showed that the extensive neuronal loss in the hippocampal areas CA1 and CA2 correlated with strong accumulation of intraneuronal Aβ, but not with extracellular Aβ deposition [189].

Furthermore, the correlation analysis shows that the increase in intracellular α-CTF has a significantly negative correlation with the age of AD onset (r = -0.661, p = 0.010), and β-CTF also shows a tendency toward a negative correlation with the age of AD onset (r = -0.341, p = 0.196) (Fig. 1). In other words, the results suggest that CTFs might cause neuronal dysfunction. Pera *et al.* found that the β-CTF level in the postmortem brain samples of EOFAD (2 cases with APP mutation and 8 cases with PS1 mutations) was significantly higher than in age-matched controls and sporadic AD. Increases in α- and β-CTF were also observed by Western blot in sporadic AD cases compared to controls [190]. Lahiri *et al.* found that immunohistochemical staining for CTFs appeared in the perinuclear region, amyloid plaques, neurites and NFT-bearing neurons, which provided evidence for the key role of CTFs in the pathogenesis of AD [191]. The level of 25-kDa CTF in cerebrospinal fluid was higher in AD with PS1 mutations, in Down syndrome, and in sporadic AD subjects [192]. The neurotoxic effects of CTF accumulation have already been reported in cellular and animal models. For example, α-CTF accumulation in Chinese hamster ovary cells could trigger impairment of the cAMP/PKA/CREB pathway (involved in synaptic plasticity and memory) without Aβ involvement [193]. The accumulation of β-CTF could induce dysfunction of endosomes in Down syndrome and AD [194]. APP C-terminal fragments AICD exerted neurotoxicity in PC12 cells and primary neurons by inducing the expression of glycogen synthase kinase 3β, causing tau phosphorylation and leading to apoptosis [195]. β-CTF was the earliest APP proteolytic product in the hippocampal neurons of 3xTg-AD mice, which suggested that β-CTF was an initiator of the neurodegenerative process in this mouse model [196]. Schettini *et al.* summarized the perturbation of the physiological activities of CTFs and AICD as an alternative perspective for neurodegeneration [197].

The ER is the main organelle involved in protein folding and secretion. During AD, the continuous accumulation of hyperphosphorylated tau and intraneuronal APP products within the ER lumen can cause chronic or irreversible ER stress, which may lead to neuronal apoptosis through unfolded protein response (UPR) [198]. Other organelle stress, including of the Golgi, mitochondrial, endosomes, and the proteasome and autophagy-lysosome, also has been linked to the intracellular accumulation of abnormal proteins and disturbance of signal pathways in neurodegenerative diseases, which could trigger neuronal cell death by a variety of endogenous suicide pathways [199-203]. Taken together, these data suggest that the intraneuronal accumulation of NFTs, Aβs, and CTFs might provoke organelle stress and lead to neurodegeneration in AD.

### An amyloid hypothesis

Hypotheses based on genetic evidence include the well-known amyloid cascade hypothesis (ACH), the presenilin hypothesis (PSH) and the more recent APP matrix approach (AMA) [8]. However, increasing evidence and questions have perforated some arguments behind these hypotheses, which will be explored. The amyloid cascade hypothesis posits the “cascade” of cellular events as stemming directly from the toxicity of amyloid beta, including NFT aggregation and neuronal loss. Many scientific findings contradict this observation, instead citing NFT aggregation or other malfunctions of processing enzymes. Specifically, AD-associated pathologies, such as amyloid plaques, can be present in individuals without any apparent cognitive impairment [204, 205]. Recent neuroimaging studies also suggested that amyloid plaques may not be directly responsible for neuronal dysfunction [206, 207]. Furthermore, neurodegeneration in AD may be independent of extracellular Aβ and oligomers, and the toxic effects observed in cellular and Tg animal models overexpressing exogenous APP may not apply to situations for most AD patients without overexpression of APP [208]. The analysis in this paper shows a weak negative correlation between increased Aβ42 production and the age of AD onset (r = -0.328, p = 0.127), which might indicate that Aβ42 production is an indirect cause of AD onset.

The presenilin hypothesis explains amyloid beta generation and the subsequent neuronal loss in terms of partial loss of function of the presenilin γ-secretase [209]. However, the majority of AD cases do not involve the dysfunction of γ-secretase; therefore, this theory is not viable to explain the general pathogenesis of AD [8, 176].

The APP matrix approach attempts to view the APP proteolytic system holistically, suggesting that a dynamic balance of APP products is necessary for proper neuronal function. It explains that although familial AD genetic mutations can alter the balance of the APP proteolytic system, other systems, e.g., cholesterol homeostasis, immune signaling, and synaptic plasticity, can alter this balance, which can lead to multiple disease pathways. Despite the scope of this hypothesis, it remains a potpourri of ideas that does not completely explain certain observations, such as how Aβ-associated pathology may be present in those without cognitive impairment.

Based on our analysis and the fact that amyloid plaque burden correlates much less well with degree of cognitive impairment than NFTs counts do, we propose the Snowball Hypothesis, a theory encompassing observed phenomena, as the ACH attempted to do with the Aβ peptide, and retaining the scope attempted by the AMA. This theory posits that the accumulation of extracellular Aβ42 does not directly cause neuronal death or dysfunction — instead, it induces stress in various protein-handling organelles such as the ER, the Golgi/TGN, and endosomes. This gradual cellular stress increases the retention of misfolded proteins, such as the hyperphosphorylated tau protein or aggregates of the α-synuclein protein. The NFTs and Lewy bodies subsequently appear in neurons depending on their susceptibility. In addition, intracellular APP proteolytic products (CTFs and Aβs) also aggravate the organelle stress. With the abnormal intracellular protein accumulation, vulnerable neurons slowly lose function and finally die, resembling abnormal protein “snowball” formations both inside and outside of neurons.

Several key questions regarding the amyloid hypothesis were raised in Selkoe and Hardy’s review paper [176]. We try to provide a perspective for answering them with the principles of the Snowball Hypothesis.

1 What are the toxic species of Aβ and tau?
  The accumulation of Aβ and tau protein occurs over a relatively long period of time, as they can be retained for two decades without noticeable cognitive decline. Therefore, we would like to say that they are an increasing burden that stresses neuronal organelles rather than being “toxic”.
2 What is the connection between Aβ and tangle pathology? Is it direct and cell autonomous or does it involve non-neuronal cells?
  Extracellular Aβ42 and intracellular APP proteolytic products (CTFs and Aβs) cause organelle stress and abnormal activation of kinases, which induce hyperphosphorylation of tau and subsequently lead to the formation of NFTs.
3 What is the mechanism of pathology spread and does understanding this spread provide therapeutic opportunities?
  The spread of pathology (mainly for NFTs) is dependent on the predisposing factors (CTFs and Aβs) and the susceptibility of neurons. Therapeutic opportunities rely on elimination of abnormal APP metabolism and relief of organelle stress in neurons.
4 What is the function of APP and does Aβ have a function?
  We believe that the major function of APP is as an adhesion molecule for synaptic formation and neurite growth. Aβs are only metabolic waste products of APP.
5 GWA studies have identified cholesterol metabolism, the innate immune system, and endosomal vesicle recycling as important pathogenic processes in AD: how do these relate to each other?
  These processes all relate to the extracellular and intracellular clearance of abnormal protein accumulation, including Aβ and hyperphosphorylated tau.

## Concluding remarks

Our summarized data from APP missense mutation studies have painted a clear picture, in which intraneuronal NFTs caused by the accumulation of extracellular Aβ42 and the increase in intraneuronal APP proteolytic products (CTFs and Aβs) could cause organelle stress in neurons, leading to neurodegeneration in AD. We must emphasize that the published clinical and neuropathological data related to APP missense mutations are incomplete. For some mutations (A673T, A673V, D678H, and I716T), only one clinical case was reported, and many mutations (D678H, D678N, E682K, K687N, E693del, V715A, V715M, I716T, I716V, L723P, and K724M) lacked neuropathological reports. The experimental protocols and reports in the cellular and Tg animal models exhibited large variations, including in the cell types, gene delivery systems, treatment procedures, methods and antibodies to investigate APP proteolytic fragments [8]. Therefore, a collection containing additional clinical cases with FAD, standardized experimental protocols for cellular and Tg animal models, and the systematic measurement of APP proteolytic fragments for all known pathogenic mutations with standardized reporting formats would help to uncover the mechanism of pathogenesis caused by APP mutations [8]. Nevertheless, this preliminary analysis still provides support for future investigation of the role of extracellular Aβ42 and intracellular APP proteolytic products. We believe that the elimination of abnormal APP proteolytic product accumulation and the relief of organelle stress for neurons would be practical treatment strategies. Secretase inhibitors, Aβ antibodies, attracting microglia to sites of Aβ accumulation [210], upregulation of proteasomes, and siRNA interference [211] also remain options. Relieving organelle stress and burden warrants further investigation. Recent developments include engineering small-molecule inhibitors of kinases such as CDK5 that can provide avenues for preventing Golgi stress and tau aggregation. Additionally, stimulating the release of intracellular Aβ localized in late endosomes or cellular compartments can prevent inappropriate interactions with organelles [212]. In general, combinations of these treatment strategies may provide the optimal methods to prevent and treat AD.
